# Comparison of diabetic retinopathy screening between hospital-based multidisciplinary and general practice-based settings: insights from a regional study in Italy

**DOI:** 10.1007/s00592-024-02354-6

**Published:** 2024-08-19

**Authors:** Chiara Olivieri, Mattia Salato, Alessandra Campanella, Paola Marolo, Guglielmo Parisi, Giovanni Neri, Mario Damiano Toro, Antonio Scarmozzino, Fabio Broglio, Enrico Borrelli, Michele Reibaldi

**Affiliations:** 1 Department of Surgical Sciences, University of Turin, Turin, Italy; 2 Department of Ophthalmology, “City of Health and Science” Hospital, Turin, Italy; 3 Medical School, University of Turin, Turin, Italy; 4 General Practice Clinic, Ciriè, Turin, Italy; 5 Public Health Department, Eye Clinic, University of Naples Federico II, 80138 Naples, Italy; 6 Department of Quality and Cure Safety, Città Della Salute E Della Scienza, Turin, Italy; 7 Division of Endocrinology, Diabetes and Metabolism, Department of Medical Sciences, University of Turin, Corso Dogliotti 14, 10126 Turin, Italy

**Keywords:** Diabetic retinopathy, Screening, Settings

## Abstract

**Purpose:**

To compare diabetic retinopathy screening among patients with type 1 or type 2 diabetes under care in two distinct setups: hospital-based multidisciplinary and general practice-based.

**Materials and methods:**

In this retrospective observational case series, we collected data from a total of 133 diabetic patients: subjects from the hospital-based multidisciplinary setting were referred by the diabetologist and screened by an ophthalmologist using the Optomed Aurora IQ fundus camera. These patients were compared with those who underwent DR screening arranged through a general practice-based setting.

**Results:**

The proportion of patients treated with insulin was higher in the hospital-based multidisciplinary group, both considering the totality patients and those affected by type 2 diabetes (71.6% vs. 32.2%; *p* < 0.001, and 58.8% vs. 31.0%; *p* = 0.004 respectively). Patients from the hospital-based multidisciplinary group had a longer mean diabetes duration (19.6 vs 14.9 years, *p* < 0.001), underwent DR screening more frequently in the previous three years (2.9 vs 1.4, *p* < 0.001), the mean time between two DR screenings was shorter (14.6 vs 77.9 weeks, *p* < 0.001), and DR was detected more frequently (32,4% vs 13.5%; *p* = 0.011).

**Conclusion:**

We were able to demonstrate that patients screened in the multidisciplinary center, which had characteristics predisposing to a higher risk of DR, were more likely to be diagnosed with DR on time, with a higher mean number of DR screenings and a shorted interval between diabetic and ophthalmological assessments.

## Introduction

According to the 10th edition of the IDF Diabetes Atlas, the estimated global prevalence of diabetes was 10.5% in 2021, impacting 537 million adults aged between 20 and 79 years [1]. Approximately 45% of diabetes cases remain undiagnosed, with a disproportionately higher proportion in low-income countries, where the number of diabetic patients is expected to increase, this partly due to their aging populations [2]. Diabetes accounts for 12.2% of global deaths across all causes and significantly influences total healthcare expenses globally [3].

Individuals with diabetes should access medical care through an integrated team-based approach, fostering a collaborative partnership between patients and physicians [4]. This approach aims to prevent or postpone complications including nephropathy, retinopathy, neuropathy, and cardiovascular issues. Diabetic retinopathy (DR) is a microvascular complication which represents the leading cause of blindness among working-age adults in high income countries. Its occurrence is associated with the duration of diabetes, chronic hyperglycemia, the presence of nephropathy, and hypertension [5–7].

Additionally, in a recent study has shown the useful role of serum levels of different types of miRNA as an useful biomarker for the early detection of DR since some miRNAs regulate the insulin secretion and play an important role in the pathophysiology of DR [8].

Given that DR represents an important public health concern, and considering the availability of a straightforward, safe, and validated screening test (i.e., retinal photography) along with effective treatments, this complication meets all criteria for screening [9].

The Professional Practice Committee (PPC) of the American Diabetes Association (ADA) advises eye examinations for retinopathy screening within five years following a diagnosis of type 1 diabetes and promptly after the diagnosis of type 2 diabetes, since these patients generally have had years of undiagnosed disease. Follow-up examinations should be conducted annually thereafter [4, 10]. Screening for DR focuses on identifying microvascular retinal alterations. Detecting these changes is highly significant as it could prompt adjustments in systemic treatment or the initiation of ocular therapies. Screening for DR can be performed through fundus examination using either direct or indirect ophthalmoscopy or slit lamp biomicroscopic examination or fundus photography. It must be emphasized that novel imaging techniques, such as structural optical coherence tomography (OCT) and OCT angiography (OCTA), exhibit high sensitivity in detecting early vascular changes [11–13]. However, their use is still not included in DR screening.

In Western countries, despite the widespread occurrence of DR, only 60 to 65% of diabetes patients undergo annual screening examinations [14, 15]. Multiple studies have found that lower educational attainment, lower income, belonging to a minority racial group, recent immigration, living in rural areas, and lacking health insurance are associated with significantly lower rates of DR screening [16–23]. Importantly, there are notable challenges involved in organizing screenings for DR. Firstly, diabetic patients and their caregivers must attend multiple appointments to assess the presence of diabetes-related systemic complications. This significant commitment may limit their ability to participate in all scheduled screening examinations within the designated timeframe. Secondly, there is a shortage of experienced healthcare professionals capable of accurately grading the severity of DR. Thirdly, achieving effective collaboration among various specialists is not always accomplished, potentially delaying the timely referral for each specialized assessment.

The Italian healthcare system is a complex mix of public and private healthcare. The Italian Government allocates funding that encompasses a universal public health insurance scheme aimed at offering complimentary or subsidized healthcare services. These services cover a range of treatments provided by healthcare professionals including general practitioners and specialists. Most DR screenings in Italy are conducted by ophthalmologists, both in private clinics and public healthcare facilities. Typically, patients are referred to these screenings by general practitioners and diabetologists. Nevertheless, there are hospital-based multidisciplinary setups where patients have the option to undergo both diabetologist assessment and DR screening within the same facility, either on the same day or with only a few days between appointments.

The objective of this study is to provide a basic overview of the distinctions in DR screening between a hospital-based multidisciplinary setup and general practice-based setting in a region located in Northern Italy.

## Methods

The Institutional Review Board (IRB) of University of Turin was notified about this retrospective observational case series.

For inclusion in the study, patients were required to have either type 1 or type 2 diabetes. Those included in the analysis had undergone at least two DR screening visits, with the most recent one being considered for analysis. Exclusion criteria consisted of: (i) diagnosis of gestational diabetes; (ii) a history of prior ocular treatment for DR; (iii) presence of DR prior to the latest DR screening; and (iv) the presence of concomitant ocular diseases.

The subjects analyzed in this study were drawn from two distinct DR screening setups: (i) hospital-based multidisciplinary, and (ii) general practice-based.

In the hospital-based multidisciplinary setting (located at the San Giovanni Antica Sede (SGAS) Hospital, part of the "City of Health and Science" Hospital in Turin, Italy), patients with a diabetes diagnosis are referred and regularly monitored by diabetologists. Within this setup, an ophthalmologist is available for DR screening twice a week, exclusively for patients referred by diabetologists within the same center. This arrangement allows for DR screening to be conducted on the same day as the diabetology assessment or shortly thereafter. During the screening process, the ophthalmologist utilizes the Optomed Aurora IQ fundus camera after administering tropicamide for dilation. Subsequently, the ophthalmologist reviews the captured images to identify any signs of DR. If DR signs are detected, the patient is then referred to the Medical Retina Service of the Ophthalmology Department at the “City of Health and Science” Hospital in Turin, Italy. All consecutive diabetic patients who met the inclusion and exclusion criteria who were screened between November 2023 and December 2023 were retrospectively analyzed (i.e., 74 patients).

Patients from the hospital-based multidisciplinary setting were compared with diabetic patients who underwent DR screening arranged through a general practice-based setting within the same geographical area. Out of approximately 1,500 patients receiving care in this setting, 98 patients had a diagnosis of diabetes mellitus, and 59 of them met the inclusion and exclusion criteria, ultimately being included in the analysis.

The following variables were collected and incorporated into the analysis: age, gender, type and duration of diabetes, therapy for diabetes, time elapsed since the last DR screening assessment, and time between the last diabetes assessment and DR screening.

## Data analysis and statistics

To assess for deviations from normal distribution, the Shapiro–Wilk test was applied to all variables. Mean values and standard deviations (SD) were calculated for each quantitative variable. Comparisons between groups were made using student T-test. Fischer’s exact test was employed to compare categorical variables. A multivariate regression analysis was conducted to explore the factors that primarily influenced a positive screening, with the presence of DR at the screening as the dependent variable.

All statistical analyses were carried out with the Jamovi software (version 2.4.12.0), setting the threshold for statistical significance at *p* < 0.05.

## Results

### Patients’ characteristics

A total of 133 diabetic patients (i.e., 74 from the hospital-based multidisciplinary setup and 59 from the general practice-based setting) were included in this analysis. Mean ± SD age of participants was 62.7 ± 15.6 years and 70.7 ± 11.6 in the two groups, respectively (*p* < 0.001). Males and females were similarly represented in the two groups (*p* = 0.467) (Table 1).

**Table 1:** Characteristics of patients included in the analysis

Characteristics	Groups		*p* value
	Patients from the hospital-based multidisciplinary setup (n = 74)	Patients from the general practice-based setup (n = 59)	
Age (years)	62.7 ± 15.6	70.7 ± 11.6	< 0.001^a^
Gender (males)	46 (62.2%)	33 (55.9%)	0.467^b^
Diabetes (type 1)	23 (31.1%)	1 (1.7%)	< 0.001^b^
Patients under insulin therapy	53 (71.6%)	19 (32.2%)	< 0.001^b^
Type 2 diabetic patients under insulin therapy	30/51 (58.8%)	18/58 (31.0%)	0.004
Duration of diabetes (years)	19.6 ± 12.4	14.9 ± 9.6	0.019^a^
Number of DR screenings in the last 3 years, mean ± SD	2.9 ± 0.8	1.4 ± 1.1	< 0.001^a^
Time between last diabetic assessment and DR screening (weeks)	14.6 ± 14.5	77.9 ± 97.0	< 0.001^a^
Presence of DR	24 (32.4%)	8 (13.5%)	0.011^b^

^a^Independent-samples T test

^b^Fischer’s exact test

Quantitative values are expressed in mean ± SD. Qualitative values are reported as number (percentage)

n: number of patiens; *DR* diabetic retinopathy

**Fig. 1:**
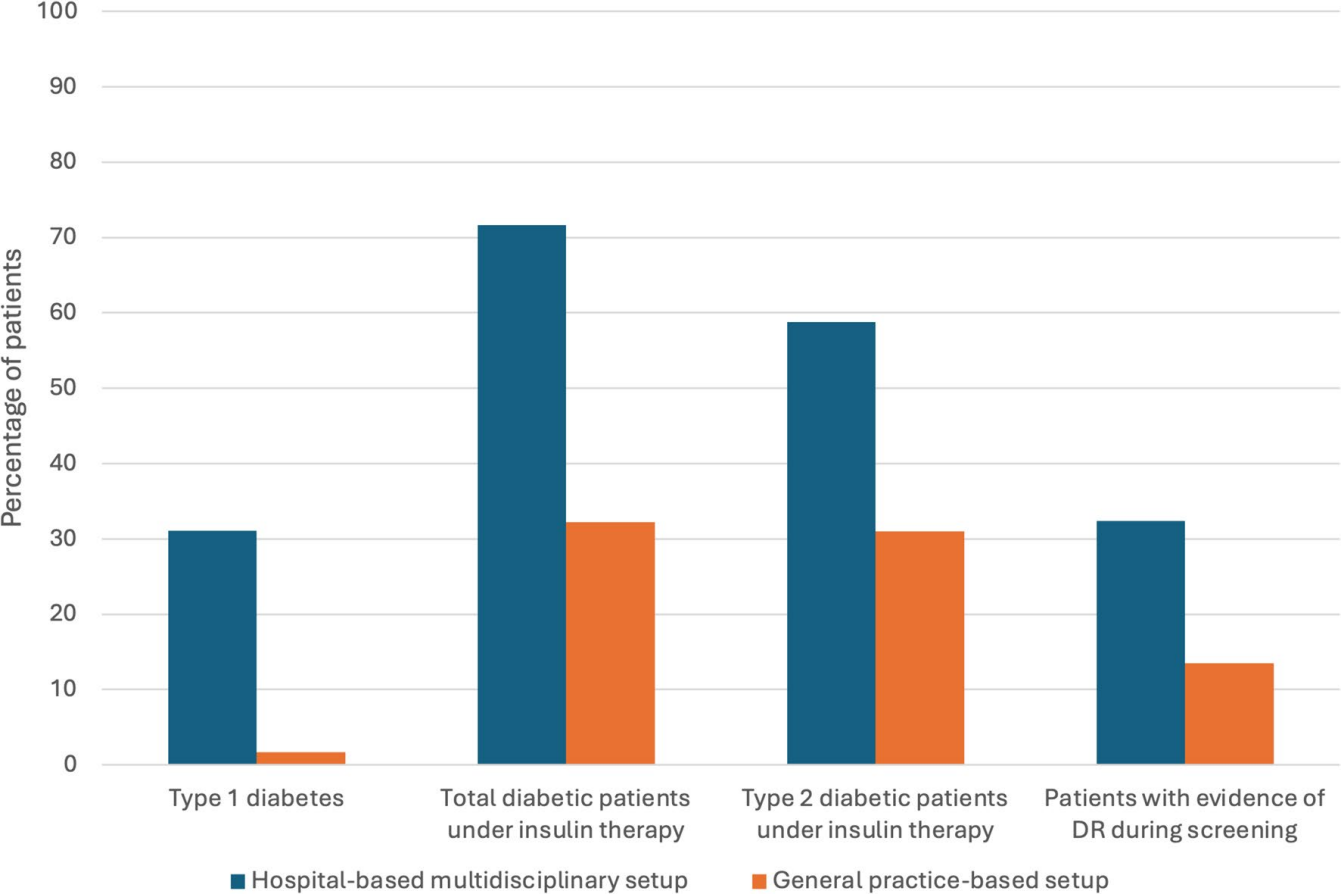
Grouped column chart showing the relative frequencies of qualitative clinical characteristics in the study cohort. Each chart shows the relative frequencies of patients with a specific clinical characteristic. The relative frequencies are given as a percentage of patients with a specific qualitative finding in a distinct group (patients from a hospital-based multidisciplinary setup vs. general practice-based setting). *P* values for each comparison are reported in the figure and details are presented in Table 1

**Fig. 2:**
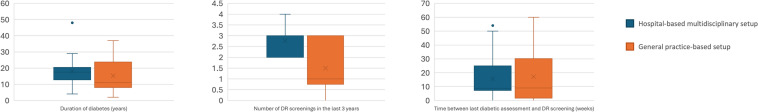
Box and whisker plots showing quantitative variables in diabetic patients. Each box displays mean (cross within the box), median (central horizontal line) and interquartile range (horizontal extremes of the box) values for each metric. The ends of the whiskers illustrate the minimum and maximum values. Outliers are visualized as dots not included in whiskers. Each graph reports comparisons for a specific quantitative characteristic. Details on comparisons are presented in Table 1

Twenty-three out of 74 patients in the hospital-based multidisciplinary group were affected by type 1 diabetes, while only 1 patient in the general practice-based setting had a diagnosis of type 1 diabetes. The percentage of patients treated with insulin was higher in the hospital-based multidisciplinary group (71.6% vs. 32.2%; *p* < 0.001), even considering only patients with type 2 diabetes (58.8% vs. 31.0%; *p* = 0.004) (Table 1, Fig. 1). Duration of diabetes was 19.6 ± 12.4 years in the hospital-based multidisciplinary group and 14.9 ± 9.6 years in the general practice-based setting group (*p* < 0.001) (Table 1, Fig. 2).

### Diabetic retinopathy screening’s characteristics

Mean ± SD number of DR screening in the last 3 years was 2.9 ± 0.8 and 1.4 ± 1.1 in the hospital-based multidisciplinary and general practice-based settings, respectively (*p* < 0.001). The time between last diabetic assessment and DR screening was 14.6 ± 14.5 weeks for patients under care in the hospital-based multidisciplinary setting and 77.9 ± 97.0 weeks for patients under care in the general practice-based setting (*p* < 0.001). Diabetic patients with a first diagnosis of DR during the latest screening were more prevalent in the hospital-based multidisciplinary group, as compared with those under care in the general practice-based setting (32.4% vs. 13.5%, *p* = 0.011) (Table 1, Fig. 2).

Among the 59 patients under care in the general practice-based setting, 5 had their most recent diabetic retinopathy screening conducted at a private practice.

The multivariate regression analysis revealed that disease duration (*p* = 0.006) and insulin therapy (*p* = 0.005) were the primary factors associated with the detection of DR during the screening (Table 2).

**Table 2:** Results of multivariable analysis with presence of diabetic retinopathy as the dependent variable

	Standardized Estimate	*P* value
Demographic and clinical factors		
Age	0.004	0.921
Type of setting (hospital-based multidisciplinary vs. general practice-based)	0.077	0.241
Type of diabetes (1 vs. 2)	0.142	0.185
Disease duration	0.004	0.006
Therapy with insulin (yes vs. no)	0.081	0.005

## Discussion

In this study, we provided a basic overview of the distinctions in DR screening features between a hospital-based multidisciplinary setup and a general practice-based setup within a region situated in Northern Italy. Overall, our findings revealed differences in the populations served by these specific settings, particularly in terms of the type and duration of diabetes. Importantly, individuals receiving care in the hospital-based multidisciplinary setup demonstrated a greater likelihood of undergoing timely and suitable DR screening compared to those referred from general practice. Moreover, patients undergoing DR screening in a multidisciplinary setting were more inclined to receive a diagnosis of DR during the ophthalmology screening, underscoring the importance of timely screening in this cohort.

As mentioned above, as per the guidelines established by the PPC AND ADA, individuals diagnosed with type 1 diabetes should undergo eye examinations for DR screening within five years of diagnosis, while those with type 2 diabetes should undergo screening promptly after diagnosis. Subsequent follow-up examinations should be conducted annually thereafter [4, 10]. The purpose of screening for DR is to identify cases requiring timely full ophthalmic examination and treatment to prevent permanent visual impairment. In our study cohort of patients undergoing DR screening through a hospital-based multidisciplinary setup, we found that the average number of DR screenings conducted in the last three years was 2.88. The latter results underscore the appropriateness of DR screening in accordance with the recommended guidelines, indicating a commendable adherence to the screening protocols.

The most relevant risk factors for the development of DR are the duration of diabetes, a diagnosis of type 1 diabetes and poor glycemic control [15, 24–27]. In our study, we observed that diabetic individuals receiving care in the hospital-based multidisciplinary setup were more inclined to have type 1 diabetes and had a longer duration of diabetes compared to those patients for whom DR screening was organized through a general practice-based setup. Moreover, within the hospital-based multidisciplinary setup, diabetic patients were more commonly undergoing treatment with insulin, even considering only individuals with type 2 diabetes. This underscores the observation that even in cases of type 2 diabetes, the proportion of our study cohort’s patients undergoing insulin treatment is higher compared to those in the general practice-based setup. The aforementioned observations collectively suggest that individuals within the study cohort who are at higher risk of developing diabetes-related complications, such as type 1 diabetic patients with a longer disease duration or type 2 diabetic patients undergoing insulin treatment, are the ones undergoing DR screening organized through a hospital-based multidisciplinary setup.

Consistently with the above mentioned findings, a higher percentage of patients undergoing DR screening through a hospital-based multidisciplinary setup exhibited signs of DR (i.e., 32.4% compared to 13.5% in our study cohort). In cases where DR is present, a prompt ophthalmologic assessment is recommended to prevent further deterioration of vision. Within the hospital-based multidisciplinary setup, the average time between the last diabetic assessment and DR screening is approximately 14 weeks, a duration significantly shorter than that observed in the general practice-based setup. This aspect is crucial, underscoring the importance of promptly referring individuals to ophthalmologists in cases where DR is detected.

While a multidisciplinary setup proves effective in conducting DR screening, it does come with limitations. First, these setups are often centralized, requiring patients and caregivers to travel long distances to access them. However, optimal outcomes are achieved when screening is offered at locations and times that match the needs of the patient, not the provider [28, 29]. In this regard, a general practice-based setup offers advantages, as it is more widespread and does not necessitate significant travel for patients. Second, implementing annual screening for all individuals with diabetes, regardless of their risk of DR, is evidently challenging to deliver and sustain within a multidisciplinary setup due to the restricted number of healthcare providers available in this environment [15]. Hence, conducting DR screening via a general practice-based setup is indispensable. However, the latter setting has limitations. For instance, there may be insufficient infrastructure to conduct screening efficiently, and patients might have to visit private ophthalmologists for screening, incurring personal expenses, or experiencing longer wait times compared to a multidisciplinary setup.

Several European studies have reported that extending the screening interval from annually to every 2 or 3 years in patients with diabetes who initially show no evidence of retinopathy can be cost-effective [30, 31]. However, in these cases it is extremely important to differentiate patients into low-risk and high-risk groups, as doing so has the potential to further enhance cost-effectivenes [32, 33]. In our study cohort, it appears that this risk stratification is observed, as patients undergoing DR screening through a general practice-based setup tend to have a lower risk of diabetes complications. This is evident as most of these patients have short-duration type 2 diabetes and are not receiving insulin treatment. Furthermore, the low incidence of signs of DR observed during screening further supports this observation.

A more effective use of appropriate digital retinal imaging coupled with telemedicine to transmit images is expected to substantially transform DR screening and enhance its effectiveness. Incorporating telemedicine into a general practice-based setup for DR screening would offer several advantages, including the ability to provide screening promptly and conveniently at locations and times that align with patient needs. This approach could be particularly beneficial for patients at low risk of DR, potentially improving DR screening outcomes in this population.

This study has certain limitations that need to be acknowledged. Firstly, while representative, the study only included two examples of multidisciplinary and general practice-based setups, which might limit the generalizability of the findings. Additionally, the study relied on DR screening results documented in patient’s records to determine screening frequency and other variables. Consequently, there is a possibility that some patients may have undergone screening, but if it was not recorded, it could not be accounted for or included in the analysis. Importantly, the diabetes care model examined in the Piedmont region is labeled as “integrated management,” dividing diabetic patients between hospital-based multidisciplinary and general practice-based setups. The latter setup mainly manages more complex cases, with general practitioners responsible for conducting retinography every two years on less complex cases. This undoubtedly influenced certain findings, potentially leading to notable variations in other regions across Italy.

In conclusion, the present study provided a basic overview of the distinctions in DR screening features between a hospital-based multidisciplinary setup and a general practice-based setup within a region situated in Northern Italy. Our findings indicate an effective DR risk stratification between these two settings, with patients at elevated risk of DR more commonly seen in the hospital-based multidisciplinary setting. However, the general practice-based setup presents advantages that could be enhanced with the implementation of telemedicine.
